# High‐Frequency Oscillatory Ventilation Combined With Inhaled Nitric Oxide for Persistent Pulmonary Hypertension of the Newborn: A Single‐Center Randomized Clinical Trial

**DOI:** 10.1155/carj/8717590

**Published:** 2026-09-28

**Authors:** Yanbo Wang, Qian Wu, Xin Zhang, Zhongyi Sun, Mingming Zhang, Xiao Liu, Bin Zhou

**Affiliations:** ^1^ Department of Pediatrics, Xuzhou Central Hospital, Xuzhou, China, xzch.cn; ^2^ Department of Nuclear Medicine, Xuzhou Central Hospital, Xuzhou, China, xzch.cn

**Keywords:** extracorporeal membrane oxygenation, high-frequency oscillatory ventilation, inhaled nitric oxide, newborn, persistent pulmonary hypertension

## Abstract

**Background:**

Persistent pulmonary hypertension of the newborn (PPHN) may respond incompletely to inhaled nitric oxide (iNO) when poorly recruited lung limits delivery to ventilated alveoli. We evaluated whether high‐frequency oscillatory ventilation (HFOV) combined with iNO improved clinical and physiologic outcomes compared with conventional mechanical ventilation (CMV) + iNO or HFOV alone.

**Methods:**

In this prospective, single‐center, 3‐arm, open‐label randomized clinical trial, 138 neonates born at 34 weeks of gestation or later with echocardiographically confirmed PPHN and an oxygenation index of 25 or greater were randomized 1:1:1 to HFOV + iNO, CMV + iNO, or HFOV alone. The principal complete‐case per‐protocol analysis included 126 infants (42 per group) with ascertainable 120‐day primary outcomes who completed the assigned strategy without a major protocol deviation. The possible influence of the 12 unavailable primary outcomes was evaluated in post hoc randomized‐denominator extreme‐case sensitivity analyses. The primary outcome was death or extracorporeal membrane oxygenation (ECMO) within 120 days.

**Results:**

In the complete‐case per‐protocol population, death or ECMO occurred in 3 of 42 infants (7.1%) assigned to HFOV + iNO, 11 of 42 (26.2%) assigned to CMV + iNO, and 14 of 42 (33.3%) assigned to HFOV alone (overall *p* = 0.012). For HFOV + iNO versus CMV + iNO, the risk ratio was 0.27 (95% CI, 0.08–0.91), and the risk difference was −19.0 percentage points (95% CI, −34.5 to −3.6; Holm‐adjusted *p* = 0.038). For HFOV + iNO versus HFOV alone, the risk ratio was 0.21 (95% CI, 0.07–0.69) and the risk difference was −26.2 percentage points (95% CI, −42.4 to −9.9; Holm‐adjusted *p* = 0.011). The mean oxygenation index at 72 h was 7.1 (SD, 2.8), 11.0 (SD, 3.8), and 15.9 (SD, 4.5), respectively (group‐by‐time interaction *p* < 0.001). The overall primary comparison remained significant under symmetric extreme‐case assignments for the unavailable outcomes but not under the differential worst‐case assumption for HFOV + iNO (*p* = 0.222).

**Conclusions:**

In the observed per‐protocol population, HFOV + iNO was associated with fewer deaths or ECMO events and faster improvement in oxygenation and right‐ventricular echocardiographic measures than either comparator. Because 12 randomized infants were excluded and the overall comparison was not robust to the differential worst‐case missing‐outcome assumption, confidence in the apparent treatment effect is limited. These findings are exploratory and hypothesis‐generating, do not establish efficacy, and require confirmation in a prospectively registered multicenter trial with complete outcome ascertainment.

## 1. Introduction

Persistent pulmonary hypertension of the newborn (PPHN) is characterized by failure of the normal postnatal decline in pulmonary vascular resistance, with right‐to‐left shunting and severe hypoxemia [1]. Observational studies in very preterm and term or near‐term populations have documented substantial mortality and practice variation [2, 3]. Multicenter and registry data further show that etiology, management, and response to inhaled nitric oxide (iNO) differ across neonatal populations [4, 5]. iNO selectively vasodilates ventilated regions of the lung and reduces the composite outcome of death or extracorporeal membrane oxygenation (ECMO) in term and near‐term neonates, although mortality alone is not consistently reduced and incomplete response remains common [6–8].

High‐frequency oscillatory ventilation (HFOV) supports gas exchange with small tidal‐volume excursions and can promote lung recruitment [9, 10]. Neonatal studies have evaluated oscillatory support across preterm and near‐term respiratory failure [11, 12]. The physiologic rationale for combining HFOV with iNO is that recruitment may reduce intrapulmonary shunt and increase the ventilated vascular surface exposed to iNO, whereas iNO acts through endothelial nitric oxide signaling to reduce extrapulmonary right‐to‐left shunting [13, 14]. A recent PPHN review supports this complementary lung‐recruitment and pulmonary‐vasodilation framework [15]. Prior trials largely evaluated rescue or crossover use rather than a parallel‐group comparison of initial strategies [16, 17]. Broader pediatric pulmonary hypertension guidance also emphasizes age‐specific phenotyping and etiologic context when interpreting treatment response [18, 19].

We therefore compared first‐line HFOV + iNO with conventional mechanical ventilation (CMV) + iNO and HFOV alone in neonates with severe, echocardiographically confirmed PPHN. The primary objective was to compare death or ECMO within 120 days. Secondary objectives were to characterize oxygenation, pulmonary pressure and right‐ventricular function, circulating biomarkers, duration of support, and acute complications. Because the trial was open‐label and single‐center, secondary physiologic and mechanistic outcomes were considered supportive and exploratory.

## 2. Methods

### 2.1. Study Design and Ethics

This prospective, single‐center, 3‐arm, open‐label randomized clinical trial was conducted at Xuzhou Central Hospital from January 2023 through June 2025. The institutional review board approved the study before enrollment (2023‐XZ‐23184), and written informed consent was obtained from at least 1 parent or legal guardian. The trial was conducted in accordance with the Declaration of Helsinki and Good Clinical Practice [20]. Reporting follows the CONSORT 2025 statement [21]. The trial was not prospectively registered; consequently, prespecification of outcomes and analyses cannot be independently verified against a public registration record, which is a substantive interpretive limitation.

### 2.2. Participants

Eligible neonates had a gestational age of at least 34 weeks, a postnatal age of 7 days or less, echocardiographic evidence of PPHN (estimated pulmonary artery systolic pressure greater than two‐thirds of systemic systolic pressure or right‐to‐left or bidirectional shunting through the ductus arteriosus or foramen ovale), and an oxygenation index of at least 25 on 2 consecutive arterial blood gas measurements while receiving an FiO2 of at least 0.80 [22, 23]. Exclusion criteria were congenital diaphragmatic hernia, cyanotic congenital heart disease other than patent ductus arteriosus or patent foramen ovale, lethal chromosomal or genetic abnormalities, severe perinatal asphyxia, birth weight below 2000 g, uncontrolled seizures, or an immediate requirement for ECMO.

### 2.3. Randomization and Masking

Participants were assigned 1:1:1 to CMV + iNO, HFOV + iNO, or HFOV alone using a computer‐generated sequence prepared before recruitment. The assignment was released only after eligibility and written consent had been confirmed, so the enrolling clinical team did not have access to future assignments. The archived analytic records did not preserve the identities of the individual who generated the sequence or the individual who released assignments. The ventilation strategy and iNO administration could not be masked. Echocardiographic measurements and laboratory assays were interpreted without access to group labels whenever feasible. Because treating clinicians were aware of assignment, ventilator adjustment, rescue decisions, ECMO initiation, and liberation from support remained susceptible to performance bias. The study was therefore open‐label and did not fully implement a formal prospective randomized open‐blinded end‐point design with systematically blinded outcome adjudication [24].

### 2.4. Interventions

HFOV was delivered with a SensorMedics 3100A or Stephanie ventilator. Initial frequency was 8–10 Hz, mean airway pressure was generally set 2–4 cm H2O above the preceding CMV mean airway pressure, and amplitude was adjusted to visible chest‐wall oscillation [9]. Recruitment was individualized using oxygenation, systemic perfusion, chest expansion, radiography, and serial echocardiography. Mean airway pressure was reduced when hyperinflation, hypotension, impaired venous return, or worsening right‐ventricular loading was suspected. This strategy reflects the U‐shaped relation between lung volume and pulmonary vascular resistance: Recruitment toward functional residual capacity may improve ventilation‐perfusion matching, whereas overdistension may compress alveolar capillaries and increase right‐ventricular afterload [25, 26].

iNO was started at 20 ppm. Response was assessed after 30 min; a brief increase to 40 ppm was permitted for nonresponse, followed by stepwise weaning after improvement in oxygen requirement. Methemoglobin was monitored, consistent with neonatal iNO practice guidance [27]. CMV was delivered in pressure‐controlled or volume‐targeted modes with lung‐protective targets [28]. Surfactant could be administered for documented or strongly suspected surfactant deficiency; combined surfactant and iNO has previously been evaluated in neonatal PPHN [29]. Sildenafil and milrinone were clinician‐directed options for refractory pulmonary hypertension [30–32], whereas vasoactive support followed the infant’s hemodynamic condition [33, 34]. Participant‐level timing and exposure to surfactant, sildenafil, milrinone, and vasoactive support were not systematically captured in the locked analysis dataset; therefore, their distribution across groups and potential contribution to the observed outcomes could not be evaluated.

### 2.5. Rescue Treatment and ECMO

Rescue crossover to HFOV + iNO was permitted for persistent severe hypoxemia despite optimization of the assigned strategy. Participants who crossed over were excluded from the principal per‐protocol analysis; their possible outcomes were represented in randomized‐denominator sensitivity analyses according to their original assignment. The locked efficacy dataset did not preserve the timing of crossover or an ascertainable 120‐day primary outcome for either of the 2 crossover participants; therefore, separate as‐treated or time‐dependent crossover analyses were not possible. ECMO was considered for potentially reversible cardiorespiratory failure with persistent severe hypoxemia despite maximal assigned and rescue therapy, generally an oxygenation index of approximately 40 or greater, after review of major contraindications, consistent with the center’s adaptation of neonatal ECMO guidance [35]. ECMO candidacy was not adjudicated by an independent masked committee; it was determined jointly by the treating neonatology and ECMO teams according to institutional practice. Because ECMO delivery is influenced by clinician judgment, expertise, and local resources, the ECMO component was not interpreted as a purely physiologic endpoint [36].

### 2.6. Outcomes

The primary outcome was death from any cause or initiation of ECMO within 120 days after randomization. Secondary outcomes were serial oxygenation index and PaO_2_/FiO_2_ ratio; estimated pulmonary artery systolic pressure, tricuspid annular plane systolic excursion, right‐ventricular to left‐ventricular basal diameter ratio, and Tei index; B‐type natriuretic peptide, endothelin‐1, cyclic guanosine monophosphate, and interleukin‐6; individual components of the primary outcome; and durations of mechanical ventilation, intensive care, hospitalization, and supplemental oxygen. Acute safety outcomes were grade III–IV intraventricular hemorrhage, pneumothorax, Bell stage II or greater necrotizing enterocolitis, stage III or greater retinopathy of prematurity, and hearing impairment. Bronchopulmonary dysplasia was omitted because no single definition was appropriate across this predominantly term and late‐preterm cohort, whereas much of the iNO evidence focused on bronchopulmonary dysplasia prevention concerns extremely preterm infants [37].

### 2.7. Statistical Analysis

The sample‐size calculation required 42 analyzable infants per group, and the recruitment target was increased to 46 per group to allow for approximately 10% attrition. A total of 138 infants were randomized, 46 to each group. Reaudit of the locked participant‐level efficacy dataset identified 126 infants (42 per group) with an ascertainable 120‐day primary outcome who completed the assigned strategy without a major protocol deviation. The principal efficacy analysis is, therefore, described as a complete‐case analysis of the per‐protocol population rather than a conventional intention‐to‐treat analysis. The 12 randomized infants excluded from this analysis had no ascertainable 120‐day primary‐outcome record in the locked dataset after major protocol deviation, parental withdrawal, equipment failure, or rescue crossover. No participant‐level outcomes were created or singly imputed. Model‐based multiple imputation was not used because the missingness mechanism could not be established and a missing‐at‐random assumption could not be justified. To quantify the possible influence of the unavailable outcomes, post hoc randomized‐denominator extreme‐case sensitivity analyses retained 46 infants in each originally assigned group under 4 assumptions: all unavailable outcomes were nonevents; all were events; all 4 unavailable HFOV + iNO outcomes were events, while all 8 unavailable comparator outcomes were nonevents; and the converse differential best‐case assumption. These analyses assess robustness and do not constitute a fully observed intention‐to‐treat estimate.

The overall 3‐group primary comparison used the Pearson chi‐square test. The 2 planned comparisons of HFOV + iNO with each comparator used 2‐sided Fisher exact tests with Holm adjustment and were summarized with risk ratios and absolute risk differences with 95% confidence intervals. Secondary categorical outcomes were compared using Pearson chi‐square tests or Fisher–Freeman–Halton exact tests when expected cell counts were small. Continuous variables are reported as mean (SD) or median (IQR), according to distribution. Repeated measurements were analyzed with linear mixed‐effects models containing group, time, and group‐by‐time interaction as fixed effects and participant as a random intercept; markedly skewed outcomes were log transformed. Duration outcomes used the Kruskal–Wallis test. A post hoc etiologic analysis summarized events within meconium aspiration syndrome, respiratory distress syndrome, idiopathic PPHN, and sepsis/pneumonia strata; homogeneity across strata was assessed separately for each HFOV + iNO comparison using the Breslow–Day test with a continuity correction. Because of sparse cells and low event counts, this analysis was considered exploratory. Secondary‐outcome *p* values were not adjusted across the complete family and are exploratory. Analyses were independently rerun from the participant‐level dataset using Python 3.13, SciPy 1.17, and statsmodels 0.14.6. All tests were 2‐sided with alpha = 0.050, and statistical reporting follows the SAMPL recommendations [38].

## 3. Results

### 3.1. Participant Flow and Baseline Characteristics

Between January 2023 and June 2025, 203 neonates were assessed for eligibility, and 65 were excluded before randomization: 41 did not meet eligibility criteria, 18 parents declined consent, and 6 were excluded for other reasons. A total of 138 infants were randomized, with 46 assigned to each group. The complete‐case per‐protocol efficacy population comprised 126 infants, 42 per group. Twelve randomized infants were not included in the principal analysis: 5 because of major protocol deviations, 4 after parental withdrawal, 1 because of equipment failure, and 2 after rescue crossover. For the 2 rescue crossovers, neither crossover timing nor the 120‐day primary outcome was recoverable. The contemporaneous flow record did not preserve whether parental withdrawal referred only to study treatment or also to continued data collection; however, no ascertainable 120‐day primary outcome for these infants was available in the locked efficacy dataset. All 138 randomized infants remained represented in randomized‐denominator extreme‐case sensitivity analyses according to their originally assigned group (Figure 1). Baseline characteristics of the per‐protocol population are summarized descriptively in Table 1.

**FIGURE 1:**
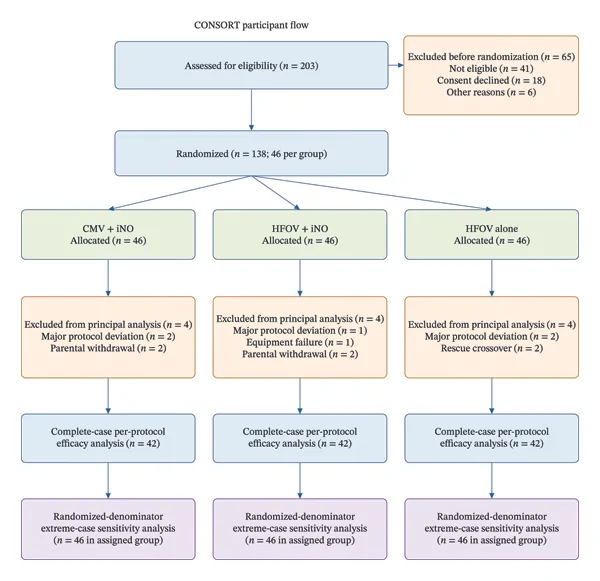
CONSORT participant flow. A total of 138 infants were randomized, with 46 assigned to each group. The principal complete‐case per‐protocol analysis included 126 infants (42 per group). Recorded postrandomization exclusions were major protocol deviation, parental withdrawal, equipment failure, and rescue crossover. The locked efficacy dataset did not contain ascertainable 120‐day primary outcomes for the 12 excluded infants. Their possible influence was evaluated in randomized‐denominator extreme‐case sensitivity analyses retaining all 46 infants in each originally assigned group. CMV indicates conventional mechanical ventilation; HFOV, high‐frequency oscillatory ventilation; iNO, inhaled nitric oxide.

**TABLE 1:** Baseline demographic and clinical characteristics.

Characteristic	CMV + iNO (*n* = 42)	HFOV + iNO (*n* = 42)	HFOV alone (*n* = 42)	*p*
GA (weeks)	38.4 ± 1.8	38.5 ± 1.6	38.9 ± 1.7	0.293
Birth weight (g)	3271 ± 217	3290 ± 224	3320 ± 226	0.597
Male sex, *n* (%)	25 (59.5)	19 (45.2)	20 (47.6)	0.374
Cesarean delivery, *n* (%)	30 (71.4)	24 (57.1)	23 (54.8)	0.238
Apgar 1 min	5.0 ± 1.8	5.4 ± 1.8	4.9 ± 1.7	0.387
Apgar 5 min	6.7 ± 2.0	6.8 ± 2.0	6.7 ± 2.0	0.992
Age at enrolment (h)	10.2 ± 6.4	12.7 ± 13.2	10.7 ± 10.8	0.519
Etiology, *n* (%)				0.651
MAS	8 (19.0)	11 (26.2)	15 (35.7)	
RDS	14 (33.3)	11 (26.2)	8 (19.0)	
Idiopathic PPHN	11 (26.2)	11 (26.2)	12 (28.6)	
Sepsis/pneumonia	9 (21.4)	9 (21.4)	7 (16.7)	
Baseline OI	40.3 ± 10.8	42.1 ± 9.2	40.6 ± 9.6	0.658
Baseline PaO_2_ (mm Hg)	38.7 ± 9.3	37.9 ± 8.3	37.9 ± 6.4	0.865
Baseline SpO_2_ (%)	79.3 ± 6.8	77.2 ± 7.5	79.3 ± 6.3	0.308
Baseline PASP (mm Hg)	61.6 ± 12.1	61.8 ± 9.0	61.4 ± 12.8	0.987

*Note:* Values are mean (SD) or number (percentage). Baseline *p* values are presented descriptively and should not be interpreted as tests of successful randomization.

Abbreviations: MAS meconium aspiration syndrome; OI, oxygenation index; PASP, pulmonary artery systolic pressure; PPHN, persistent pulmonary hypertension of the newborn; and RDS, respiratory distress syndrome.

### 3.2. Oxygenation

Oxygenation improved in all groups, with the largest and most rapid reduction in oxygenation index in the HFOV + iNO group (group‐by‐time interaction *p* < 0.001) (Table 2; Figure 2). At 72 h, the mean oxygenation index was 7.1 (SD, 2.8) with HFOV + iNO, 11.0 (SD, 3.8) with CMV + iNO, and 15.9 (SD, 4.5) with HFOV alone. The corresponding mean differences for HFOV + iNO were −3.9 (95% CI, −5.3 to −2.4) versus CMV + iNO and −8.7 (95% CI, −10.4 to −7.1) versus HFOV alone. The PaO2/FiO2 ratio was right‐skewed and was summarized as median (IQR); the log‐scale group‐by‐time interaction was *p* < 0.001 (Table 2; Figure 3).

**TABLE 2:** Serial oxygenation measures.

Time	CMV + iNO	HFOV + iNO	HFOV alone	Statistic	*p*
OI					
Baseline	40.3 ± 10.8	42.1 ± 9.2	40.6 ± 9.6	0.42	0.658
1 h	33.0 ± 9.4	30.1 ± 7.1	35.8 ± 9.3	4.62	0.012
4 h	27.6 ± 7.4	23.1 ± 5.7	30.0 ± 7.2	11.18	< 0.001
12 h	22.5 ± 6.6	17.4 ± 4.5	26.8 ± 7.2	24.15	< 0.001
24 h	18.2 ± 5.7	12.7 ± 3.9	22.3 ± 6.0	35.03	< 0.001
48 h	13.7 ± 4.5	8.9 ± 3.4	18.0 ± 4.1	53.14	< 0.001
72 h	11.0 ± 3.8	7.1 ± 2.8	15.9 ± 4.5	57.04	< 0.001
PaO_2_/FiO_2_					
Baseline	48.4 (39.1–58.1)	47.7 (39.7–57.6)	42.1 (32.5–53.6)	2.12	0.346
24 h	102.2 (78.2–136.1)	152.2 (127.4–187.6)	80.5 (56.5–97.3)	51.82	< 0.001
72 h	172.4 (134.6–214.0)	265.2 (205.0–383.4)	109.6 (81.8–145.7)	65.58	< 0.001

*Note:* OI values are mean (SD), and PaO_2_/FiO_2_ values are median (IQR). Overall *p* values are from 1‐way analysis of variance for OI and Kruskal–Wallis tests for PaO_2_/FiO_2_ at each time point. Longitudinal inference was based on mixed‐effects models; PaO_2_/FiO_2_ was log transformed.

**FIGURE 2:**
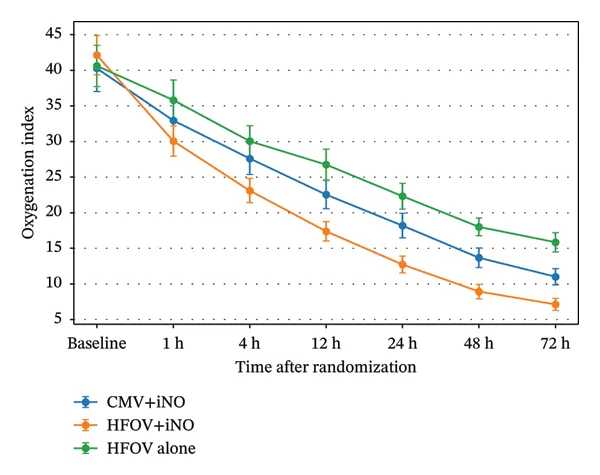
Oxygenation index through 72 h in the complete‐case per‐protocol population (*n* = 42 per group). Points are means, and error bars are 95% confidence intervals. The group‐by‐time interaction was *p* < 0.001. OI indicates oxygenation index; CMV, conventional mechanical ventilation; HFOV, high‐frequency oscillatory ventilation; and iNO, inhaled nitric oxide.

**FIGURE 3:**
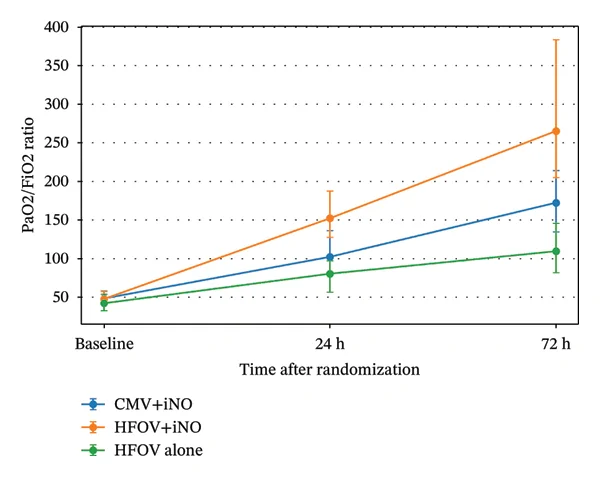
PaO2/FiO2 ratio through 72 h in the complete‐case per‐protocol population (*n* = 42 per group). Points are medians, and error bars are interquartile ranges. Longitudinal analysis used log‐transformed values; the group‐by‐time interaction was *p* < 0.001. CMV indicates conventional mechanical ventilation; HFOV, high‐frequency oscillatory ventilation; and iNO, inhaled nitric oxide.

### 3.3. Echocardiographic and Biomarker Outcomes

Estimated pulmonary pressure and right‐ventricular indices improved most in the HFOV + iNO group (Table 3). At 72 h, mean PASP was 25.9 (SD, 5.2) mm Hg with HFOV + iNO, 34.1 (SD, 7.7) mm Hg with CMV + iNO, and 39.2 (SD, 8.8) mm Hg with HFOV alone (Figure 4). Group‐by‐time interactions were *p* < 0.001 for PASP, TAPSE, RV/LV ratio, and Tei index. These secondary measures remain susceptible to measurement and performance bias in an open‐label trial.

**TABLE 3:** Echocardiographic measures.

Parameter	CMV + iNO	HFOV + iNO	HFOV alone	F	*p*
PASP (mmHg)					
Baseline	61.6 ± 12.1	61.8 ± 9.0	61.4 ± 12.8	0.01	0.987
24 h	45.2 ± 10.1	36.0 ± 6.1	48.3 ± 10.7	20.05	< 0.001
72 h	34.1 ± 7.7	25.9 ± 5.2	39.2 ± 8.8	34.56	< 0.001
TAPSE (mm)					
Baseline	5.7 ± 1.5	5.9 ± 1.3	5.7 ± 1.5	0.27	0.764
24 h	8.0 ± 2.1	10.3 ± 2.3	7.1 ± 1.9	24.02	< 0.001
72 h	10.2 ± 2.4	13.0 ± 1.7	8.8 ± 2.3	41.77	< 0.001
RV/LV ratio					
Baseline	1.21 ± 0.17	1.26 ± 0.16	1.24 ± 0.17	0.95	0.388
24 h	0.87 ± 0.13	0.74 ± 0.11	0.99 ± 0.13	41.62	< 0.001
72 h	0.66 ± 0.11	0.54 ± 0.06	0.80 ± 0.14	56.09	< 0.001
Tei index					
Baseline	0.53 ± 0.07	0.54 ± 0.09	0.55 ± 0.08	0.72	0.489
24 h	0.38 ± 0.05	0.32 ± 0.05	0.44 ± 0.07	48.33	< 0.001
72 h	0.28 ± 0.04	0.22 ± 0.04	0.36 ± 0.06	102.82	< 0.001

*Note:* Values are mean (SD). Overall *p* values are from 1‐way analysis of variance at each time point. Longitudinal inference was based on mixed‐effects models.

**FIGURE 4:**
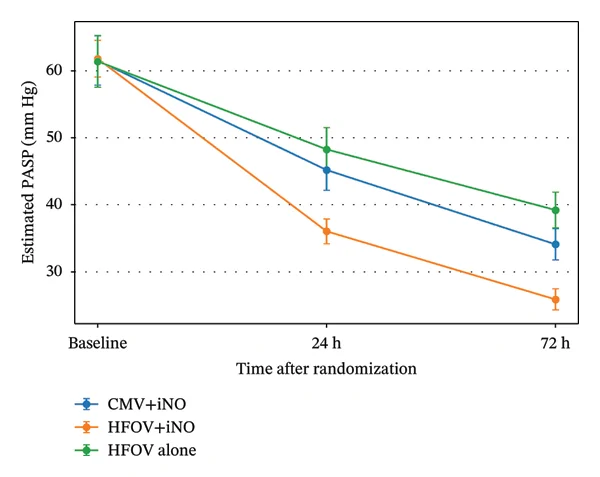
Estimated pulmonary artery systolic pressure at baseline, 24 h, and 72 h in the complete‐case per‐protocol population (*n* = 42 per group). Points are means, and error bars are 95% confidence intervals. The group‐by‐time interaction was *p* < 0.001. CMV indicates conventional mechanical ventilation; HFOV, high‐frequency oscillatory ventilation; iNO, inhaled nitric oxide; and PASP, pulmonary artery systolic pressure.

Biomarker trajectories also favored HFOV + iNO (Table 4). At 72 h, mean cyclic guanosine monophosphate was 114.7 (SD, 39.0) pmol/mL with HFOV + iNO, 88.8 (SD, 24.2) pmol/mL with CMV + iNO, and 62.0 (SD, 16.4) pmol/mL with HFOV alone. B‐type natriuretic peptide and interleukin‐6 were right‐skewed and are summarized as median (IQR).

**TABLE 4:** Biomarker measurements.

Biomarker	CMV + iNO	HFOV + iNO	HFOV alone	Statistic	*p*
BNP (pg/mL)					
Baseline	475.0 (348.2–879.0)	548.5 (381.8–800.2)	573.5 (474.5–816.2)	0.54	0.765
24 h	334.0 (210.0–569.8)	280.5 (183.5–395.2)	459.0 (355.5–607.5)	13.98	< 0.001
72 h	233.0 (173.5–400.0)	178.5 (108.2–277.2)	370.0 (257.0–451.2)	23.14	< 0.001
ET‐1 (pg/mL)					
Baseline	24.5 ± 6.5	26.0 ± 5.8	24.7 ± 6.5	0.68	0.507
24 h	15.9 ± 4.7	12.8 ± 3.7	18.9 ± 5.2	18.26	< 0.001
72 h	11.1 ± 4.0	7.9 ± 3.0	14.9 ± 4.0	37.59	< 0.001
cGMP (pmol/mL)					
Baseline	37.9 ± 9.1	35.1 ± 8.0	36.2 ± 8.3	1.14	0.322
24 h	58.5 ± 14.4	71.2 ± 17.9	46.8 ± 11.9	28.04	< 0.001
72 h	88.8 ± 24.2	114.7 ± 39.0	62.0 ± 16.4	36.79	< 0.001
IL‐6 (pg/mL)					
Baseline	49.8 (30.8–82.4)	53.5 (24.2–84.6)	53.0 (30.6–100.3)	0.11	0.944
24 h	40.0 (19.1–51.1)	26.3 (13.9–44.9)	40.1 (25.3–69.1)	6.02	0.049
72 h	22.9 (13.5–39.4)	13.8 (6.3–23.8)	30.0 (18.7–67.7)	17.62	< 0.001

*Note:* ET‐1 and cGMP are mean (SD); BNP and IL‐6 are median (IQR). Overall *p* values are from 1‐way analysis of variance or Kruskal–Wallis tests, as appropriate. Longitudinal models used log transformation for BNP and IL‐6.

### 3.4. Primary and Clinical Outcomes

In the complete‐case per‐protocol population, death or ECMO occurred in 3 of 42 infants (7.1%) with HFOV + iNO, 11 of 42 (26.2%) with CMV + iNO, and 14 of 42 (33.3%) with HFOV alone (overall *p* = 0.012) (Table 5). For HFOV + iNO versus CMV + iNO, the risk ratio was 0.27 (95% CI, 0.08–0.91), the risk difference was −19.0 percentage points (95% CI, −34.5 to −3.6), and the Holm‐adjusted *p* value was 0.038. For HFOV + iNO versus HFOV alone, the risk ratio was 0.21 (95% CI, 0.07–0.69), the risk difference was −26.2 percentage points (95% CI, −42.4 to −9.9), and the Holm‐adjusted *p* value was 0.011 (Table 6). The corresponding numbers needed to treat were 5.2 and 3.8, but estimates were imprecise because only 28 composite events occurred. In randomized‐denominator extreme‐case sensitivity analyses, the overall comparison remained significant when all 12 unavailable outcomes were assigned as nonevents (11/46, 3/46, and 14/46; *p* = 0.013) and when all were assigned as events (15/46, 7/46, and 18/46; *p* = 0.033). Under the differential worst‐case assumption for HFOV + iNO, the corresponding counts were 11/46, 7/46, and 14/46, and the overall comparison was no longer significant (*p* = 0.222). The converse differential best‐case scenario remained significant (*p* < 0.001) (Table 7).

**TABLE 5:** Clinical outcomes and acute complications.

Outcome	CMV + iNO (*n* = 42)	HFOV + iNO (*n* = 42)	HFOV alone (*n* = 42)	*p*
Primary outcome				
Death or ECMO, *n* (%)	11 (26.2)	3 (7.1)	14 (33.3)	0.012
Components				
Death, *n* (%)	4 (9.5)	2 (4.8)	6 (14.3)	0.389
ECMO, *n* (%)	7 (16.7)	1 (2.4)	8 (19.0)	0.033
Duration outcomes				
MV (d), median (IQR)	9.9 (7.0–12.6)	6.7 (5.7–9.0)	11.4 (8.8–15.4)	< 0.001
ICU stay (d)	13.8 (9.6–18.1)	11.9 (8.1–15.1)	16.8 (12.3–22.1)	< 0.001
Hospital stay (d)	19.6 (14.2–26.3)	16.7 (10.9–20.6)	21.1 (17.1–26.4)	0.003
O_2_ supplementation (d)	12.4 (9.1–14.8)	10.4 (7.5–13.2)	14.4 (10.8–18.5)	< 0.001
Complications, *n* (%)				
IVH (grade III–IV)	3 (7.1)	3 (7.1)	2 (4.8)	1.000
Pneumothorax	4 (9.5)	3 (7.1)	5 (11.9)	0.928
NEC (≥ stage II)	2 (4.8)	2 (4.8)	1 (2.4)	1.000
ROP (≥ stage III)	2 (4.8)	2 (4.8)	1 (2.4)	1.000
Hearing impairment	2 (4.8)	1 (2.4)	1 (2.4)	1.000

*Note:* Binary outcomes are numbers (percentage), and durations are medians (IQR). The overall primary comparison used the Pearson chi‐square test; sparse secondary categorical outcomes used the Fisher–Freeman–Halton exact test, and duration outcomes used the Kruskal–Wallis test. The study was not powered for mortality alone, uncommon complications, or safety equivalence.

**TABLE 6:** Pairwise effect estimates for selected outcomes.

Outcome	HFOV + iNO vs. CMV + iNO	HFOV + iNO vs. HFOV alone
Death or ECMO	RR 0.27 (95% CI, 0.08–0.91); RD −19.0 points (95% CI, −34.5 to −3.6); adjusted *p* = 0.038	RR 0.21 (95% CI, 0.07–0.69); RD −26.2 points (95% CI, −42.4 to −9.9); adjusted *p* = 0.011
OI at 72 h	Mean difference −3.9 (95% CI, −5.3 to −2.4)	Mean difference −8.7 (95% CI, −10.4 to −7.1)
PaO2/FiO2 at 72 h	Geometric mean ratio 1.71 (95% CI, 1.40–2.09)	Geometric mean ratio 2.70 (95% CI, 2.21–3.29)
PASP at 72 h	Mean difference −8.2 mm Hg (95% CI, −11.1 to −5.4)	Mean difference −13.3 mm Hg (95% CI, −16.5 to −10.2)
TAPSE at 72 h	Mean difference 2.8 mm (95% CI, 1.9–3.7)	Mean difference 4.2 mm (95% CI, 3.3–5.1)
cGMP at 72 h	Mean difference 25.9 pmol/mL (95% CI, 11.8–40.1)	Mean difference 52.7 pmol/mL (95% CI, 39.6–65.8)

Abbreviations: OI, oxygenation index; PASP, pulmonary artery systolic pressure; RD, risk difference; RR, risk ratio; TAPSE, tricuspid annular plane systolic excursion; and cGMP, cyclic guanosine monophosphate.

**TABLE 7:** Randomized‐denominator extreme‐case sensitivity analyses for death or ECMO.

Assumption for 12 unavailable outcomes	CMV + iNO	HFOV + iNO	HFOV alone	Statistical result
All unavailable outcomes assigned as nonevents	11/46	3/46	14/46	Overall *p* = 0.013; Holm‐adjusted *p* = 0.039 and *p* = 0.012
All unavailable outcomes assigned as events	15/46	7/46	18/46	Overall *p* = 0.033; Holm‐adjusted *p* = 0.086 and *p* = 0.036
Differential worst case for HFOV + iNO	11/46	7/46	14/46	Overall *p* = 0.222; Holm‐adjusted *p* = 0.431 and *p* = 0.270
Differential best case for HFOV + iNO	15/46	3/46	18/46	Overall *p* < 0.001; Holm‐adjusted *p* = 0.003 and *p* < 0.001

*Note:* Values are events/46 randomized infants. Pairwise Holm‐adjusted *p* values are listed in the order HFOV + iNO versus CMV + iNO and HFOV + iNO versus HFOV alone. These post hoc analyses retain all randomized participants under transparent extreme‐case assignments and are robustness analyses, not substitutes for a fully observed intention‐to‐treat estimate.

### 3.5. Etiology Subgroup Analysis

In the post hoc analysis, event counts by PPHN etiology were directionally favorable to HFOV + iNO in most strata but were sparse (Supporting Table S1). Breslow–Day tests did not detect heterogeneity of the association across etiologic strata for HFOV + iNO versus CMV + iNO (*p* = 0.521) or HFOV alone (*p* = 0.213). These tests had limited power, and the subgroup results should not be interpreted as evidence of a uniform treatment effect.

## 4. Discussion

In the observed complete‐case per‐protocol population, HFOV + iNO was associated with a lower incidence of death or ECMO than CMV + iNO or HFOV alone. The absolute risk differences were 19.0 percentage points versus CMV + iNO and 26.2 percentage points versus HFOV alone. The lower incidence of death or ECMO was accompanied by faster improvement in oxygenation, estimated pulmonary pressure, and right‐ventricular indices. However, this was not a fully observed intention‐to‐treat estimate. The overall comparison remained statistically significant under symmetric extreme‐case assumptions for the 12 unavailable outcomes but was no longer significant under the most unfavorable differential assumption for HFOV + iNO. Accordingly, the apparent benefit is not robust to a differential missing‐outcome assumption that cannot be excluded, and this study does not establish efficacy.

The observed physiologic changes are biologically plausible and are broadly consistent with current understanding of pulmonary vascular physiology. iNO reaches pulmonary vessels adjacent to ventilated alveoli; recruitment of atelectatic lung may therefore improve ventilation‐perfusion matching and expose a larger vascular bed to selective vasodilation through endothelial nitric oxide signaling [13, 14]. The greater increase in cyclic guanosine monophosphate and decrease in endothelin‐1 with HFOV + iNO are compatible with enhanced nitric oxide‐soluble guanylate cyclase signaling, but circulating biomarkers are downstream correlates rather than direct measures of pulmonary iNO delivery [39, 40].

Recruitment must be balanced against right‐ventricular and systemic hemodynamics. Pulmonary vascular resistance is lowest near an appropriate functional residual capacity and may increase at both low and excessive lung volumes. Excessive mean airway pressure may overdistend alveoli, compress pulmonary capillaries, reduce venous return, and increase effective right‐ventricular afterload [41, 42]. The observed pulmonary artery systolic pressure, tricuspid annular plane systolic excursion, right‐ventricular to left‐ventricular ratio, and Tei‐index trajectories suggest that the observed echocardiographic trajectories are consistent with improved cardiopulmonary adaptation, but they do not exclude transient adverse hemodynamic effects. Individualized pressure titration using oxygenation, perfusion, lung expansion, and functional echocardiography is therefore preferable to routine pressure escalation.

Systems‐level studies in adult pulmonary hypertension provide methodological context for future neonatal research but should be interpreted only as mechanistic context for treatment of PPHN. Adult pulmonary arterial hypertension reviews and text‐mining studies illustrate mechanism‐informed drug‐repurposing frameworks [43, 44]. *Trans*‐right‐ventricular metabolomics provides an example of integrated right‐ventricular and pulmonary vascular phenotyping [45]. Mendelian randomization analysis in another cardiovascular domain illustrates a strategy for causal hypothesis generation rather than direct evidence of a microbiome mechanism in PH [46]. These studies neither justify neonatal use of sodium–glucose cotransporter 2 inhibitors or other repurposed agents nor establish a neonatal microbiome treatment target; their relevance is limited to generating age‐specific hypotheses for future prospective cohorts.

Acute complication rates did not differ detectably, but nonsignificant comparisons in this sample do not establish safety equivalence. The shorter duration of mechanical ventilation may be clinically meaningful, although ventilator weaning and extubation were clinician‐directed and could have been influenced by knowledge of treatment assignment. Duration and biomarker outcomes should be interpreted as exploratory.

This study has limitations. First, the principal efficacy analysis was a complete‐case analysis of the per‐protocol population rather than a conventional intention‐to‐treat analysis. Twelve of 138 randomized infants did not have an ascertainable 120‐day primary outcome in the locked efficacy dataset. Although randomized‐denominator sensitivity analyses supported the overall result under symmetric assumptions, statistical significance was lost under the most unfavorable differential assumption for HFOV + iNO; informative postrandomization missingness and selection bias therefore remain possible, and the magnitude of the observed effect may be overestimated. Thus, both the direction and magnitude of the apparent treatment effect remain uncertain, and the complete‐case result cannot support an efficacy conclusion. Second, the open‐label design permits performance and ascertainment bias, particularly for ventilator adjustment, rescue decisions, ECMO initiation, echocardiographic measurements, and liberation from support. Third, ECMO use depends partly on institutional expertise, resources, and clinician judgment and was not adjudicated by an independent masked committee. Fourth, the exact timing and observed outcomes of the 2 recorded rescue crossovers were not recoverable from the locked efficacy dataset. Accordingly, neither an as‐treated effect nor the direction of crossover‐related bias can be determined. Fifth, the single‐center sample was small for mortality, uncommon adverse events, and treatment‐by‐etiology interactions; the post hoc etiologic analysis was underpowered and included zero‐event cells. Sixth, clinician‐directed cointerventions such as surfactant, vasoactive drugs, sildenafil, and milrinone were not systematically captured in the locked analysis dataset, so balance across groups could not be assessed. Differential cointervention use therefore cannot be excluded and may have contributed to between‐group differences, further weakening attribution of the observed outcomes to the assigned strategy. Seventh, the documentation of allocation concealment was incomplete in the archived study records, and the archived records did not distinguish whether parental withdrawal applied only to assigned treatment or also to outcome follow‐up; these details were not reconstructed. Eighth, multiple secondary outcomes were examined without family‐wise multiplicity adjustment. Ninth, long‐term neurodevelopmental follow‐up was not available, although neurosensory outcomes are clinically relevant after major neonatal cardiopulmonary interventions [47]. Finally, the trial was not prospectively registered. This prevents independent verification that the reported outcomes and analyses were prespecified and increases concern about selective outcome or analysis reporting.

In conclusion, HFOV + iNO was associated with fewer deaths or ECMO events and faster improvement in oxygenation and right‐ventricular echocardiographic measures in the observed per‐protocol population. However, exclusion of 12 randomized infants, loss of statistical significance under the differential worst‐case missing‐outcome assumption, unrecoverable crossover data, unmeasured clinician‐directed cointerventions, and absence of prospective registration substantially limit causal confidence. These findings are exploratory and hypothesis‐generating; they do not establish efficacy, safety equivalence, a preferred first‐line strategy, or a basis for guideline change. A prospectively registered multicenter trial with complete outcome ascertainment, documented allocation concealment, standardized rescue and ECMO criteria and cointerventions, prespecified heterogeneity analyses, and long‐term neurodevelopmental follow‐up is required.

## Author Contributions

Bin Zhou, the corresponding author and manuscript guarantor, had full access to all of the data in this study and takes complete responsibility for the integrity of the data and the accuracy of the data analysis.

## Funding

This study was supported by the Xuzhou Science and Technology Program Project (KC23184).

## Disclosure

The guarantor affirms that this manuscript is an honest, accurate, and transparent account of the study; that no important aspects have been omitted; and that discrepancies from the study as planned and reported have been explained. The funder had no role in study design, recruitment, data collection, analysis, interpretation, manuscript preparation, or the decision to submit. All authors have read and approved the final version of the manuscript.

## Conflicts of Interest

The authors declare no conflicts of interest.

## Supporting Information

Additional supporting information can be found online in the Supporting Information section.

## Supporting information


**Supporting Information** Supporting Table S1 presents death or ECMO event counts, denominators, and percentages by PPHN etiology and treatment group in the complete‐case per‐protocol population. The accompanying table note reports the post hoc exploratory Breslow–Day homogeneity tests and notes the limitations imposed by sparse cells.

## Data Availability

Deidentified participant‐level data and the data dictionary may be made available from the corresponding author on reasonable request, subject to institutional and ethics approval.
