# Supplementary material for: High‐Frequency Oscillatory Ventilation Combined With Inhaled Nitric Oxide for Persistent Pulmonary Hypertension of the Newborn: A Single‐Center Randomized Clinical Trial
Source: Can Respir J. 2026 Sep 28;2026:8717590. doi: 10.1155/carj/8717590 (PMC13617540; doi:10.1155/carj/8717590)
Supplement: Supplementary file 1 [file CARJ-2026-8717590-s001.docx]

Supplementary material

High-Frequency Oscillatory Ventilation Combined with Inhaled Nitric Oxide for Persistent Pulmonary Hypertension of the Newborn: A Single-Center Randomized Clinical Trial

Manuscript ID: 8717590

**Supplementary Table S1. Death or ECMO by PPHN etiology**

| **Etiology** | **CMV+iNO** | **HFOV+iNO** | **HFOV alone** |
| --- | --- | --- | --- |
| Meconium aspiration syndrome | 3/8 (37.5) | 0/11 (0) | 8/15 (53.3) |
| Respiratory distress syndrome | 3/14 (21.4) | 2/11 (18.2) | 1/8 (12.5) |
| Idiopathic PPHN | 1/11 (9.1) | 0/11 (0) | 3/12 (25.0) |
| Sepsis/pneumonia | 4/9 (44.4) | 1/9 (11.1) | 2/7 (28.6) |

Values are event counts/total infants (percentage). The analysis was post hoc and exploratory. The Breslow-Day homogeneity *p* values were 0.521 for HFOV+iNO versus CMV+iNO and *p* = 0.213 for HFOV+iNO versus HFOV alone.

Abbreviations: CMV, conventional mechanical ventilation; ECMO, extracorporeal membrane oxygenation; HFOV, high-frequency oscillatory ventilation; iNO, inhaled nitric oxide; PPHN, persistent pulmonary hypertension of the newborn.
